# Supplementary material for: The in vitro assessment of rheological properties and dentin remineralization of saliva substitutes containing propolis and aloe vera extracts
Source: PLoS One. 2024 May 22;19(5):e0304156. doi: 10.1371/journal.pone.0304156 (PMC11111055; doi:10.1371/journal.pone.0304156)
Supplement: Attachment — Submitted filename: Response to reviewers.pdf [file pone.0304156.s002.pdf]

Dear Reviewers,

Thank you for dedicating your time to reviewing our manuscript and for providing valuable feedback. We greatly appreciate your constructive comments and suggestions, which have guided our revisions and enhanced the quality of our work. We have carefully reviewed and incorporated your comments and suggestions into the revised manuscript (changes have been highlighted in red).

Please find attached a table providing point-by-point responses to each of your comments. Your expertise and insights have been invaluable to us.

Sincerely,

Dr. Piyaphong Panpisut DDS, MSc, PhD

Associate Professor in Dental Material Science

Faculty of Dentistry, Thammasat University
99 Moo18, T. Klong Nueng, A. Klong Luang
Pathum Thani, 12120 Thailand
Email: panpisut@tu.ac.th
Tel: +66 891613189

Comment from reviewer 1	
**Comment 1**	**The type of study (animal study, in vitro, or in vivo study) would be appreciated in the title of the article. Preclinical studies, such as in vitro trials, help us measure drug efficacy before administering a medicine to humans. The problem with them, however, is that there is a big difference between a drug administrated in vitro and the same drug administered in a human, so this information is important in the title, and its clinical relevance should be carefully interpreted at the beginning.**
Answer	We agree with the reviewer that it is essential for the title and abstract to clearly indicate the specific type of study to prevent confusion among readers. Accordingly, we have revised both the title and abstract to reflect that this is an *in vitro* study.
Revision	• Title: The *In Vitro* Assessment of Rheological Properties and Dentin Remineralization by Saliva Substitutes Containing Propolis and Aloe Vera Extracts<br>• Abstract: Modifications made at Line 23
**Comment 2**	**The main conclusion of the article should be state on the abstract instead of "These promising properties could be beneficial for the prevention of dental caries in xerostomic patients" (line 38-39).**
Answer	We sincerely thank the reviewer for the comment, which has helped us improve the clarity of the abstract. We have revised the conclusion to more directly state the significant findings and implications of our research
Revision	Abstract Lines 38-40 "To conclude, the saliva substitute containing propolis extract demonstrated superior remineralizing actions compared with those containing only aloe vera extract."
**Comment 3**	**The text is consistent; it contains sufficient and organized information about the topic to its purpose. However, some information should be addressed.**<br>**62-63: "The commercially available prescribed saliva substitute has limited specific therapeutic effects, such as mineralization and antimicrobial actions." What are the available options? Reference studies that proved its limitations.**

Comment from reviewer 1	
Answer	After careful consideration, we agree with the reviewer regarding the lack of supporting evidence to substantiate the claim about the limitations of commercially available saliva substitutes. Consequently, we have removed this statement from the manuscript to ensure all information presented is evidence-based and accurately referenced.
Revision	Line 68
**Comment 4**	**Furthermore, some given information is not referenced. For example: the sentence from line 51-52 and 80-81; Please add references, even if the authors are talking about the reference exposed at the end of the paragraph, after each phrase it should be exposed. Multiple in-text citations to the same work over a large section of text can be visually jarring and it is true that they are not entirely necessary. However, the rule of thumb is to cite the very first sentence, make it clear you are still talking about the same work in your subsequent sentences (for example, "The study noted that..."), and then confirm you are still talking about the work by including another citation at the end (if this has continued for several sentences).If you have a simple follow-on sentence in which it is still clear that you are talking about the same work, you do not need the reference in the second sentence. If at any point you think it might not be clear in the sentence that you are still referring to the same work, include another in-text citation, otherwise, it will appear as an information without a proper citation.**
Answer	We appreciate the invaluable suggestions from the reviewer regarding citation practices. We have reviewed our citation style and revised the manuscript accordingly to ensure clear attribution of sources throughout the text.
Revision	Lines 69-88
**Comment 5**	**96-97: The authors should at least summarize how the artificial saliva was performed in the text, even if described in another paper, you should briefly describe how it was performed in your article and then reference the source.**
Answer	We sincerely thank the reviewer for this helpful suggestion. To clarify the methodology, we have now included a brief summary of the artificial saliva preparation process in the Materials and Methods section of our manuscript.
Revision	Lines 119-123

Comment from reviewer 1	
**Comment 6**	**134-135: Again, explain how it was performed. What is the difference from previous studies? Why? Overall, the information in this section is very condensed, even though you have to summarize the steps performed, all the information should be stated to ensure reproducibility.**
Answer	We sincerely thank the reviewer for emphasizing the importance of detailed methodological descriptions to ensure reproducibility. In response, we have expanded the descriptions in the methods section, detailing each step to clarify the step for the reader.
Revision	Lines 165-172
**Comment 7**	**234: The aim of the study is already stated on the introduction section and it should not be here again. Some statements also require references. The text also would benefit from a discussion with more studies addressed to discuss the findings of the present study.**
Answer	We appreciate your observations regarding the redundancy and referencing within our manuscript. We have removed the repeated mention of the study's aim as suggested. Additionally, we have enriched the discussion section by incorporating more references that support and help elucidate our findings. However, we must note that there is a limited number of original research studies that have conducted similar or relevant experiments, which constrains our ability to make extensive comparisons
Revision	Lines 349-368

Comment from reviewer 2	
**Comment 1**	**The percentage mentioned, forecasting the increase in the proportion of elderly individuals from 12% to 22% by 2050, is derived from the WHO's Ageing and Health report, and is not attributed to AlQranei et al. (2020) [1]. For accurate sourcing, please refer to the following link: https://www.who.int/news-room/fact-sheets/detail/ageing-and-health. Accessed on March 24, 2020.**
Answer	We appreciate the reviewer for highlighting the need to correct our citation source. We acknowledge this oversight and have accordingly updated the citation to accurately reflect that the data regarding the increase in the elderly population comes from the WHO's Ageing and Health report, as recommended.
Revision	Lines 46-47
**Comment 2**	**The prevalence of dental caries, particularly root caries, among the elderly is undoubtedly a significant oral health concern. However, to ensure the currency and robustness of the information presented, it is imperative to incorporate more recent statistics or references regarding this issue. Additionally, it's crucial to recognize that the percentage cited, originating from Lopez et al. (2017), is based on a review article primarily focused on studies conducted in the USA and other developed countries. Consequently, there may be limitations in extrapolating these findings to developing countries, where there could be potential variations across different demographics or geographical regions.**
Answer	We greatly appreciate the reviewer's insights, which have highlighted the need for a broader geographic perspective in our study. In response, we have updated the manuscript to include more recent data on the prevalence of root caries in developing countries.
Revision	Lines 48-50
**Comment 3**	**The chemical formula and mechanism of action of propolis and aloe vera extracts are essential aspects to elucidate in your manuscript. Could you kindly include detailed explanations of their chemical compositions and mechanisms of action? This information will enhance the understanding of their therapeutic potential and contribute to the comprehensiveness of your study.**

Comment from reviewer 2	
Answer	Thank you for emphasizing the importance of detailing the chemical compositions and mechanisms of action of propolis and aloe vera extracts. We recognize the significance of these details in understanding the therapeutic potential of these substances. Accordingly, we have expanded the relevant sections of the manuscript to include information on the typical chemical constituents of propolis and aloe vera, as well as their proposed therapeutic mechanisms based on current scientific understanding.
Revision	Lines 69-78, 82-83
**Comment 4**	**While the introduction of your manuscript provides an overview of the techniques utilized to assess rheological characteristics, such as strain-controlled rheometer and compact-DDM method, it lacks an exploration of potential limitations or challenges associated with these techniques. For instance, it would be beneficial to address concerns regarding the accuracy, consistency, and comparability of measurements obtained using the compact-DDM method, particularly in laboratory settings. Please consider further examination of these aspects to provide a comprehensive understanding of the methodology employed in your study.**
Answer	Thank you for your insightful comments. We have revised the introduction part to include the potential limitations and challenges associated with the compact-DDM method as suggested by the reviewer.
Revision	Lines 95-110
**Comment 5**	**It is essential to acknowledge the potential variability in individual responses to experimental saliva substitutes, which may not have been fully addressed in the introduction of your manuscript. Considering the diverse responses among individuals, it would be beneficial to discuss this aspect further to provide a comprehensive understanding of the study's implications.**
Answer	Thank you for highlighting the importance of addressing individual variability in responses to experimental saliva substitutes. We agree that understanding this variability is crucial for interpreting the broader implications of our study. Accordingly, we have revised the introduction to include findings from the recent systematic reviews that discuss the outcomes in how patients with xerostomia respond to different saliva substitutes.

Comment from reviewer 2	
Revision	Lines 62-65
**Comment 6**	**kindly include group 6 distilled water in Table 2 of your manuscript? This addition will ensure completeness and clarity in presenting the experimental groups and their respective treatments.**
Answer	Thank you for your suggestion to enhance the clarity and completeness of our presentation of experimental groups. We have added Group 6, which utilizes deionized water, to Table 2 as recommended. This update ensures that all treatment groups are accurately and comprehensively represented.
Revision	Table 2
**Comment 7**	**Could you please provide clarification on why the sample temperature was maintained at 25°C rather than at 37°C, which is the normal temperature in the oral cavity? This deviation from physiological conditions warrants explanation to ensure the relevance and applicability of your experimental setup to real-clinical scenarios.**
Answer	We sincerely thank the reviewer for this insightful question. The decision to maintain the sample temperature at 25°C, rather than the physiological norm of 37°C, was made due to equipment limitation. Specifically, while the strain-controlled rheometer can adjust for temperature, the compact-DDM, used in the experiments, does not yet have temperature control functions. This limitation and its potential impact on the results have been duly acknowledged and discussed in the manuscript to ensure clarity regarding the experimental conditions and their applicability.
Revision	Lines 333-335
**Comment 8**	**Could you kindly explain the rationale behind choosing to cut the crown of the teeth horizontally instead of perpendicularly? Given that a perpendicular cut would likely result in a greater exposure of dentinal tubules compared to a horizontal cut, I am interested in understanding the reasoning behind this decision. Your insights would be greatly appreciated.**
Answer	We appreciate the reviewer's query regarding the orientation of tooth sectioning in our study. The decision to cut the crowns horizontally was primarily to minimize the risk of including the pulp space, which could complicate the handling and analysis of the specimens. This horizontal orientation facilitates the production of dentin discs, enhancing handling and ease of manipulation for FITR assessment and surface examination

Comment from reviewer 2	
	with SEM-EDX. While this method limits our ability to observe the depth of occlusion along the dentinal tubules, it aligns with our study's primary focus, which is not on depth measurement but rather on the presence and extent of tubule occlusion
Revision	-
**Comment 9**	**Could you kindly include more details in your manuscript regarding the aperture size used during ATR-FTIR measurements, the surface treatment and polishing of the samples, as well as the dehydration process and rehydration?**
Answer	We have revised the manuscript and included protocol for cleaning of samples and FTIR study in the materials and method section
Revision	Lines 167-173, Lines 179-194
**Comment 10**	**I kindly inquire whether the phenomenon of crack formation in the samples due to the process of dehydration and rehydration has been observed in your study. Understanding any potential effects of these processes on sample integrity would contribute to the robustness of your findings.**
Answer	We appreciate the reviewer's concern regarding the potential for crack formation in our samples due to the processes of dehydration and rehydration. During our experiments, no visible cracks were observed in the specimens. This may be due to the conditions under which the cycles of demineralization and remineralization were conducted, where the specimens likely remained sufficiently moist. We acknowledge the importance of this aspect for the integrity of the findings and will consider further monitoring and reporting on these conditions in future studies to ensure the robustness of our results.
Revision	-
**Comment 11**	**1. I kindly note that the graph indicates a significant shift in the phosphate peak, which appears to be unreported. Additionally, in such cases, it is preferable to measure the area under the curve rather than focusing solely on peak height for a more comprehensive analysis.**<br>**2. Moreover, there are other important parameters worth considering to better understand the effects of propolis and aloe vera. These include the carbonate to phosphate ratio,**

Comment from reviewer 2	
	**collagen cross-linking, and the degree of crystallinity of dentin. It is evident from the chart that these parameters are also influenced to a significant extent. Integrating these aspects into your analysis would enhance the depth of understanding of your study outcomes.**
Answer	1. We thank the reviewer for this insightful comment. We were also analyzed the data using area under peak or peak height which showed the similar trend. Additionally, we acknowledge the significant shift in the phosphate peak after demineralization/remineralization and have now reported this observation in the materials and methods section, as well as discussed the possible reasons behind this finding in the discussion part.<br>2. We appreciate the suggestion to examine additional parameters such as the carbonate to phosphate ratio, collagen cross-linking, and the degree of crystallinity of apatite using XRD. While the current study focused primarily on screening remineralizing actions to aid future clinical trials or product optimizations, we recognize the value of these suggestions and have included a discussion on these future research directions in the manuscript.
Revision	1. Lines 343<br>2. Lines 387-389
**Comment 12**	**I would like to kindly point out that the study employs a simplified demineralization model involving EDTA. While this approach serves its purpose, it may not fully replicate the complex and multifactorial nature of natural caries development. Considering the diverse factors involved in caries formation, such as biofilm presence, incorporating more diverse demineralization models could enhance the clinical relevance of the remineralization test.**
Answer	We appreciate the reviewer's insightful observations regarding the use of EDTA in our demineralization model. We acknowledge that while EDTA serves as a useful simplification for controlled experimental conditions, it does not fully capture the multifaceted nature of natural caries development

Comment from reviewer 2	
	that a biofilm model might better represent. We have therefore recognized this limitation in our manuscript and expanded the discussion to address the potential benefits of employing more complex demineralization models, such as biofilm models, in future studies to increase the clinical relevance of our findings.
Revision	Lines 400-405
**Comment 13**	**I kindly note that the remineralization protocol in your study spans only 14 days, providing valuable insights into short-term effects. However, it may be beneficial to consider extending the duration of the study to assess the sustainability and long-term remineralization potential of the saliva substitutes. Longer-term studies could offer valuable insights into the durability of remineralization outcomes over extended periods, thereby enriching the comprehensiveness of your findings.**
Answer	We completely agree with the reviewer's comment that a 14-day pH cycling protocol might not represent the long-term ability of the saliva substitutes to promote remineralization. This shorter duration was chosen to facilitate the characterization of remineralizing effects among various formulations, allowing us to screening the promising candidates for further evaluation in long-term studies. We have addressed this limitation in the manuscript and highlighted the need for future investigations using extended pH cycling or biofilm models to mimic the oral environment more closely.
Revision	Lines 400-405
**Comment 14**	**Could you kindly provide information regarding the acquisition parameters used to collect the EDX data, including the kilovoltage (kV) and working distance (WD)? Additionally, I recommend calculating the ratios of calcium to phosphorus (Ca:P) and calcium to carbon (Ca:C) to further elucidate the elemental composition. Moreover, I emphasize the critical importance of thorough data analysis from SEM images in dentin samples. Considering the varied composition of different dentin areas, such as intertubular dentin and peritubular dentin, careful analysis is essential to ensure a comprehensive understanding of the findings. It would be helpful to specify from exactly where the measurements were taken to provide clarity and context to the results.**

Comment from reviewer 2	
Answer	- Thank you for your detailed inquiry. The EDX acquisition parameters are critical for accurate data interpretation, and this information was provided in the revised manuscript. Regarding the elemental composition, our analysis suggested that the majority of precipitates detected from SEM might not be calcium phosphates, but potentially silicate compounds occluding the tubules, which could hinder the detection of apatite underneath. This condition might explain the weak signals from calcium and phosphorus, rendering the calculation of a Ca:P ratio (such as the expected 1.67 for apatite) not applicable in this context.<br>- The EDX analysis was primarily used to characterize the surface precipitates observed on the dentin samples. We acknowledge the need to indicate the location of reading which is now labelled in the figures to clarify the context and enhance the understanding of our findings.
Revision	Figure 5
**Comment 15**	**I kindly note that while the results of your study suggest promising trends in terms of rheological properties and remineralization potential, it is important to consider several limitations. Specifically, the discrepancies observed in viscosity measurements between the rheometer and compact-DDM highlight potential technical challenges or limitations in accurately assessing rheological properties, particularly at higher shear rates. Acknowledging and addressing these limitations will strengthen the interpretation and applicability of your findings**
Answer	We would like to thank for the comment and included our compact-DDM limitation at higher shear rate in the discussion section.
Revision	Lines 325-342
**Comment 16**	**I kindly suggest discussing the results of remineralization in more depth, considering the chemical mechanism of remineralization of demineralized dentin. Exploring the mechanisms underlying the observed effects, including the role of specific components in propolis and aloe vera extracts, would provide valuable insights for clinical applications. Your attention to these aspects would enrich the**

Comment from reviewer 2	
	**discussion and enhance the relevance of your findings for clinical practice.**
Answer	We sincerely thank the reviewer for their suggestion, which has highlighted an opportunity to deepen the discussion of our findings. In response, we have highlighted the discussion section to explore in greater detail the chemical mechanisms of remineralization associated with demineralized dentin, particularly focusing on the roles of specific components found in propolis, which is correlate to our main findings. This enhancement includes a discussion on how these components interact at a molecular level to facilitate remineralization, which is directly relevant to the results observed in our study.
Revision	Lines 356 - 369
